# Distinct behavioral consequences of short-term and prolonged GABAergic depletion in prefrontal cortex and dorsal hippocampus

**DOI:** 10.3389/fnbeh.2014.00452

**Published:** 2015-01-13

**Authors:** Judith M. Reichel, Sabine Nissel, Gabriela Rogel-Salazar, Anna Mederer, Karola Käfer, Benedikt T. Bedenk, Henrik Martens, Rebecca Anders, Jens Grosche, Dominik Michalski, Wolfgang Härtig, Carsten T. Wotjak

**Affiliations:** ^1^Department of Stress Neurobiology and Neurogenetics, Max Planck Institute of Psychiatry, Research Group “Neuronal Plasticity”Munich, Germany; ^2^Paul Flechsig Institute for Brain Research, University of LeipzigLeipzig, Germany; ^3^Synaptic Systems GmbHGöttingen, Germany; ^4^Effigos AGLeipzig, Germany; ^5^Department of Neurology, University of LeipzigLeipzig, Germany

**Keywords:** hippocampus, prefrontal cortex, GABA, immunolesion, schizophrenia, cognition, hyperactivity

## Abstract

GABAergic interneurons are essential for a functional equilibrium between excitatory and inhibitory impulses throughout the CNS. Disruption of this equilibrium can lead to various neurological or neuropsychiatric disorders such as epilepsy or schizophrenia. Schizophrenia itself is clinically defined by negative (e.g., depression) and positive (e.g., hallucinations) symptoms as well as cognitive dysfunction. GABAergic interneurons are proposed to play a central role in the etiology and progression of schizophrenia; however, the specific mechanisms and the time-line of symptom development as well as the distinct involvement of cortical and hippocampal GABAergic interneurons in the etiology of schizophrenia-related symptoms are still not conclusively resolved. Previous work demonstrated that GABAergic interneurons can be selectively depleted in adult mice by means of saporin-conjugated anti-vesicular GABA transporter antibodies (SAVAs) *in vitro* and *in vivo*. Given their involvement in schizophrenia-related disease etiology, we ablated GABAergic interneurons in the medial prefrontal cortex (mPFC) and dorsal hippocampus (dHPC) in adult male C57BL/6N mice. Subsequently we assessed alterations in anxiety, sensory processing, hyperactivity and cognition after long-term (>14 days) and short-term (<14 days) GABAergic depletion. Long-term GABAergic depletion in the mPFC resulted in a decrease in sensorimotor-gating and impairments in cognitive flexibility. Notably, the same treatment at the level of the dHPC completely abolished spatial learning capabilities. Short-term GABAergic depletion in the dHPC revealed a transient hyperactive phenotype as well as marked impairments regarding the acquisition of a spatial memory. In contrast, recall of a spatial memory was not affected by the same intervention. These findings emphasize the importance of functional local GABAergic networks for the encoding but not the recall of hippocampus-dependent spatial memories.

## Introduction

GABAergic interneurons mediate several key features throughout the central nervous system. They are important for the equilibrium between excitatory and inhibitory inputs, which in turn is necessary for a functioning neuronal network (Markram et al., 2004; Kullmann et al., 2012). Although GABAergic interneurons represent only about one fifth of cortical neurons and even less in the dorsal hippocampus (dHPC; Jinno and Kosaka, 2010; Lehmann et al., 2012), a disturbance of GABAergic development or the GABAergic network quickly leads to detrimental consequences as seen in neurological and neuropsychiatric disorders, such as epilepsy, depression or schizophrenia (Sanacora et al., 2000; Wong et al., 2003; Hossein Fatemi et al., 2005; Inan et al., 2013; Gilani et al., 2014).

The GABA hypothesis of schizophrenia often implicates the frontal cortex as one of the key players in the disease etiology and progression (Blum and Mann, 2002). Clinically, schizophrenia is characterized by positive symptoms (e.g., hallucinations or movement disorders), negative symptoms (e.g., anhedonia) and cognitive dysfunction (e.g., deficits in working memory), which also involves the hippocampal structure (Tamminga and Holcomb, 2005; Powell and Geyer, 2007). Especially the positive symptoms are difficult to mimic in animal models; therefore, a distinction is used for endophenotypes with little face validity but high predictive validity (e.g., hyperlocomotion) or endophenotypes with face-, construct- and predictive validity, such as altered sensorimotor gating (Swerdlow and Geyer, 1998; Powell and Geyer, 2007).

There are several subclasses of GABAergic interneurons, which can be distinguished based on their morphology, their expression of calcium-binding proteins (e.g., parvalbumin, calbindin or calretinin), the co-expression of neuropeptides (e.g., somatostatin or vasointestinal peptide) and electrophysiological properties (Freund and Buzsáki, 1996; Markram et al., 2004; Kubota, 2014). Each of these subclasses has been linked to particular functions and/or distributions throughout the forebrain (Markram et al., 2004; Kubota, 2014). However, Parvalbumin-positive (PV+) interneurons, representing one of the largest GABAergic subclasses, have been especially implicated in learning and memory related neuronal plasticity (Caillard et al., 2000; Donato et al., 2013; Bissonette et al., 2014).

Cognitive tasks such as spatial memory acquisition and the recall of a spatial memory crucially depend on an intact hippocampal formation (Morris, 1984; Nakazawa et al., 2004; Schlesiger et al., 2013). Particularly the feed-forward inhibition via fast spiking interneurons in the CA3 region of the hippocampal formation has been shown to be intricately involved in the precise acquisition of a spatial or contextual memory (Ruediger et al., 2011). In contrast, reversal learning of spatial memories (i.e., cognitive flexibility) additionally relies on the prefrontal cortex (PFC; Euston et al., 2012; Ashwell and Ito, 2014; Baker and Ragozzino, 2014).

However, the hippocampus (HPC) and the PFC are not only integral for cognitive abilities in mice as well as men, but both have also been implicated in schizophrenia-related symptom development, where, in turn, cognitive deficits are recognized as a prominent symptom of the disease (Kuperberg and Heckers, 2000; Lewis, 2000; Blum and Mann, 2002; Sesack and Carr, 2002; Sweatt, 2004; Ross et al., 2006; Waltz and Gold, 2007; Euston et al., 2012; Ibrahim and Tamminga, 2012; Nakazawa et al., 2012; Savanthrapadian et al., 2013; Bissonette et al., 2014).

There are a number of techniques available to selectively inactivate and/or deplete GABAergic interneurons in order to study their effects on exploratory and cognitive behavior and their involvement in schizophrenia-related symptoms. While the optogenetic tool box offers very elegant and sophisticated approaches to discern a specific local neuronal circuit (Fenno et al., 2011; Madisen et al., 2012; Buetfering et al., 2014), here, we aimed to investigate the consequences of chronic GABAergic cell loss and thus a global dysfunction in the PFC and the dHPC. We employed the local administration of targeted saporin-conjugated anti-vesicular GABA transporter antibodies (SAVAs; Antonucci et al., 2012). SAVAs are directed against the intravesicular epitopes of the vesicular GABA transporter (VGAT). Upon fusion of the vesicles with the presynaptic membrane, these epitopes become accessible to the extracellular milieu and thereby to the conjugated antibodies (Martens et al., 2008). Subsequent recycling of the vesicles leads to internalization of the SAVAs, which then accumulate in GABAergic nerve terminals and ultimately lead to the destruction of GABAergic neurons due to saporin toxicity, i.e., ribosome inactivation (Wiley, 1992; Antonucci et al., 2012). We chose this technique in order to investigate the behavioral effects of GABAergic ablation over time in a region-specific manner by targeting either the prelimbic cortex (PrL) or the dHPC. We hypothesized that this rather broad network manipulation will resemble a pathological-like neuronal functional allostasis (McEwen, 2000) and thus reveal new insight into the pathology-related symptom development following the degeneration of GABAergic neurons. With respect to the temporal dynamics of neuropathological changes following SAVA treatment (Antonucci et al., 2012), we assessed behavioral consequences of SAVA at early (<10 days) vs. late time points (>14 days) after administration. We focused our analyses on positive symptoms (i.e., development of hyperactivity and deficits in pre-pulse inhibition (PPI) of the startle response) and cognitive impairments. More specifically, schizophrenia-related hyperactivity-like behavior was assessed via an open field (OF) test under red-light conditions (Jacob et al., 2009). Altered sensorimotor gating responses were assessed by presenting varying minor acoustic noise bursts at different intervals before the main acoustic startle impulse was presented in an acoustic startle response (ASR) protocol (Golub et al., 2009). Cognitive impairments after GABAergic depletion were investigated using a contextual fear conditioning (FC) paradigm and/or a spatial learning paradigm in a modified water maze task (Kleinknecht et al., 2012). Although negative symptoms such as alterations in social behavior are a common finding among schizophrenic patients (Girardi et al., 2014), given the relatively short time frame between selective GABAergic depletion and secondary glutamatergic neuronal loss (Antonucci et al., 2012) and the limited involvement of GABAergic neurons of the PFC in social interaction (Yizhar et al., 2011), we decided to forego social behavior-related testing and focused on hyperactivity, sensorimotor gating and cognitive consequences of GABAergic depletion.

We investigated the effects of GABAergic ablation in two different brain structures and for varying incubation periods after SAVA administration: >14 days of incubation after SAVA application were used to study long-term effects of GABAergic depletion in the PrL and dHPC, and <10 days were chosen to track short-term consequences of GABAergic interneuron depletion in the dHPC of C57BL/6N mice. In addition, we confirmed that the dHPC is involved not only in the acquisition, but also the recall of spatial memory, by local infusion of the GABA_A_ agonist Muscimol into the dHPC.

## Materials and methods

All experimental procedures were approved by the Committee on Animal Health and Welfare of the State of Bavaria (Regierung von Oberbayern, Munich, Germany, AZ 55.2-1-54-2532-142-12) and were performed in compliance with the European Economic Community (EEC) recommendations for the care and use of laboratory animals (2010/63/EU). Every effort was done to minimize animal suffering and to reduce the necessary sample sizes.

### SAVA

SAVAs have been prepared as previously described (Antonucci et al., 2012). Briefly, 1 mg reduced rabbit anti-VGAT-C (131103, Synaptic Systems, Göttingen, Germany) was coupled to 2 mg of saporin (Sigma-Aldrich, Taufkirchen, Germany) with the bifunctional cross-linker sulfosuccinimidyl 6-[α-methyl-α-(pyridyldithio) toluamido]hexanoate (sulfo-LC-SMPT, Thermo Fisher Scientific, Schwerte, Germany). Free saporin was removed by affinity purification of SAVA using VGAT-C immunogen immobilized on sulfolink coupling resin (Thermo Fisher Scientific).

### Animals

In this study a total of 109 male C57BL/6N mice from the Max Planck Institute breeding facility in Martinsried, Germany, were used. Mice were approximately 10 weeks of age at delivery and were allowed to acclimatize to the new surrounding for 10 days before surgery. After surgery animals were housed individually in type II standard Makrolon cages in a 12 h : 12 h inverse light/ dark cycle (lights off 9 a.m.) in a temperature and humidity controlled room with access to food and water *ad libitum*. All behavioral testing was performed during the dark phase between 9 a.m. and 9 p.m.

### Experimental schedules

We investigated the effects of SAVA-induced GABAergic lesions in four different experiments: **Experiment (I)** to** Experiment (IV)** and performed one additional control experiment (**Experiment (V)**) to assess the general involvement of the dHPC in spatial memory recall.

In **Experiment (I)** we targeted the PrL bilaterally at lateral (l) 0.5 (from midline); anterior-posterior (a-p) +1.9 (from Bregma); ventral (v) 2.5 mm (from the surface of the skull) with a volume of 0.5 µl each side. Ten mice were injected with phosphate-buffered saline (PBS) and 10 mice with SAVAs. Both groups were allowed to recover from surgery for 14 days before behavioral testing. On days 15 to 21 after surgery, mice underwent basal phenotyping in the OF, Dark-Light box (D-L box) and ASR test, the latter both for direct Input-Output (I/O) measurements as well as Pre-Pulse Inhibition/Facilitation (PPI/PPF). On days 22 to 36 after surgery, mice underwent cognitive testing in the Water-Cross Maze (WCM) and FC. On day 37 after surgery, mice were perfused transcardially with 4% PFA − PBS + 0.1% glutaraldehyde, and the fixed brains were harvested for histological processing.

With **Experiment (II)** we targeted the dHPC by bilateral injections at l 1.3; a-p −1.8; v 2.0 mm with a volume of 2 µl each side. We injected 8 mice with PBS, 8 mice with un-conjugated SAVAs (i.e., anti-vesicular GABA transporter antibodies without saporin; ucAB) and 8 mice with SAVAs (SAVA). All three groups were allowed to recover from surgery for 14 days before behavioral testing. Behavioral testing and final brain harvesting were done in parallel to **Experiment (I)**.

**Experiment (III)** investigated the short-term effects of SAVA injection in the dHPC on spatial memory ***acquisition***. We implanted guide cannulas bilaterally at l 1.3; a-p −1.8; v 1.0 mm, allowed the animals to recover for 12 days and only then injected 0.5 µl of either PBS (*n* = 13) or SAVA (*n* = 13) per cannula. On day 2 post injection (pi) animals underwent testing in the OF, and on day 3 to 9 pi animals were trained in the WCM before being tested in the OF once more on day 10 pi. Animals were transcardially perfused and brains harvested on day 11 pi.

**Experiment (IV)** investigated the short-term effects of SAVA injection in the dHPC on spatial memory ***recall***. We implanted cannulas as described for **Experiment (III)**, and allowed the animals to recover for 12 days. Afterwards mice were tested in the OF on day 13, trained in the WCM on day 14–day 20 followed by intra-hippocampal injections with 0.5 µl of either PBS (*n* = 10) or SAVA (*n* = 14) on day 21 post cannula implantation (= d0 pi). On day 2 and 8 pi mice were tested in the OF whereas on day 3 + 4 pi and day 9 + 10 pi spatial memory recall in the WCM was examined. Animals were transcardially perfused and brains recovered on day 11 pi.

**Experiment (V): Muscimol–Control**: In order to determine whether the dHPC was involved in the recall of a spatial memory at all, we implanted guide cannulas for a new cohort of mice as described for **Experiment (III)**, allowed the animals to recover for 12 days and then trained them in the WCM for 7 days until all mice had successfully learned the platform position. Two days later, we injected 0.125 µg muscimol dissolved in 0.5 µl saline into each hemisphere. This dose corresponds to the dosages used in the literature (Allen et al., 2008; Biedenkapp and Rudy, 2009; McEown and Treit, 2010; Oliveira et al., 2010; Stackman et al., 2012).

Ten minutes after the injection mice were again placed in the WCM and recall of spatial memory was assessed for six trials per animal. As a treatment control we employed a “within-subject design”, i.e., 24 h after the MSC test, mice were injected with PBS and again tested in the WCM for 6 trials.

### Open field

OF testing was performed under red-light conditions in order to observe pure locomotor effects rather than anxiety-related behavior (Carola et al., 2002). OF testing was performed as described previously (Jacob et al., 2009; Yen et al., 2013). Briefly, animals were placed in an OF box (26 × 26 × 38 cm, Coulbourn Instruments, Allentown, PA, USA) and allowed to explore freely for 30 min.

The floor of the box was surrounded by two infrared sensor rings in order to record horizontal and vertical movements. The infrared sensors were located 2 and 5 cm above the floor, spaced apart by 1.52 cm and connected to a computer running the Tru Scan Software Version 1.1 (Coulbourn Instruments) with a sampling rate of 4 Hz. Each OF box, was surrounded by an additional box made of opaque Plexiglas side walls (47 × 47 × 38 cm).

After testing, mice were returned to their home cages, and the OF boxes and floor planes were carefully cleaned with water and dried. If only the lower infrared beams recorded beam-brakes (all four paws on the floor), the program scored this as horizontal movement/rest, if both lower and upper rows of infrared beams recorded beam-brakes, this was scored as vertical movement (e.g., rearing), if only the upper row of beams recorded beam- brakes it was scored as jumping. Total horizontal movement (i.e., distance), frequency of vertical movements (i.e., rearing) and duration of vertical movements were later analyzed in 5 min bins.

### Dark-light box

D-L Box testing was performed as previously described (Jacob et al., 2009).

Briefly, D-L consisted of a dark compartment (15 × 20 × 25 cm) and an illuminated (600 lux) compartment (30 × 20 × 25 cm), which were connected by a 4 cm-long tunnel. Duration of testing was 5 min per animal, at the beginning of which each animal was placed in the dark compartment. The entire box was thoroughly cleaned between animals with water containing detergent and dried before placing the next animal inside. After testing, latency to enter the light compartment, frequency to enter the light compartment and relative time (duration) spent in the light compartments were scored by a trained observer blind to the animals’ treatment by means of the EVENTLOG software (designed by Robert Henderson in 1986).

### Acoustic startle response

ASR was assessed as previously described (Golub et al., 2009). In brief, mice were placed in a non-restrictive Plexiglas cylinder which was mounted onto a plastic platform located in a sound attenuated chamber (SR-LAB, San Diego Instruments, San Diego, CA, USA). This set-up quantifies changes in the conductance as a response to varying acoustic stimuli. These changes in conductance were detected by a piezoelectric sensor located underneath each cylinder. The startle amplitude was defined as the peak voltage output within the first 50 ms after stimulus onset. The startle stimuli itself consisted of 20 ms white noise bursts at 75, 90, 105 and 115 dB SPL against a constant background noise of 50 dB SPL and the startle response Input/Output curve was assessed *via* a protocol consisting of 136 pseudo-randomized trials of aforementioned white noise bursts. All cylinders were thoroughly cleaned with water containing detergent between animals. Mean startle amplitude per stimulus intensity was later analyzed in an Input/Output curve.

PPI/PPF was assessed within the same set-up as the ASR, but with a different protocol. During this protocol animals were presented with a brief pre- pulse white noise burst of 55, 65 or 75 dB SPL intensity at varying Inter Pulse Intervals (IPI) of either 5, 10, 25, 50 or 100 ms before the acoustic stimulus, a 50 ms white noise burst of 115 dB SPL. This protocol consisted of 270 pseudo-randomized trials and mean startle amplitude per pre-pulse intensity was calculated by subtracting the startle amplitude at the 115 dB test pulse from the startle amplitude after pre-pulse presentation, and dividing this by the startle amplitude at 115 dB × 100.

### Fear conditioning

FC was performed as previously described (Kamprath and Wotjak, 2004). In brief, mice were placed in conditioning chambers (ENV-307A, Med Associates Inc., St. Albans, VT, USA) with elongated Plexiglas walls and a grid floor for shock application. The grid floor was placed above bedding identical to the home cage bedding. The conditioning context was thoroughly cleaned and sprayed with 70% ethanol (EtOH) before placing each mouse inside. On day 0 of the FC protocol mice were placed in the conditioning context and left to explore it for 3 min under house light (0.6 Lux) conditions. After these 3 min a 20 s tone (9 kHz at 80 dB SPL) was presented, the last 2 s of which co-terminated with a 0.7 mA foot shock. After shock application mice remained in the conditioning context for an additional 60 s without tone presentation before being placed back to their home cage. The conditioning context and the novel context were additionally placed in separate sound attenuating isolation boxes. CCD cameras inside each isolation box allowed for behavioral scoring after testing.

On day 1 a.m. mice were placed back in the conditioning context for 3 min under house light conditions without tone or shock presentations. On day 1 p.m. mice were placed in a novel context with different contextual features (e.g., Cylinder instead of cubicle, bedding without grid, 1% acetic acid (CH_3_COOH) instead of EtOH) under house light conditions for 3 min without tone presentation followed directly by a 3 min tone presentation.

After testing, freezing behavior (i.e., immobility except for breathing) was scored *via* EVENTLOG software (designed by Robert Henderson in 1986) by a trained observer blind to the animals’ treatments.

### Water cross maze

WCM training was performed using the hippocampus-dependent place learning protocol (Kleinknecht et al., 2012). In brief, the WCM consisted of four arms termed N, E, S, W made of transparent Plexiglas. It was filled with water (22°C ± 1°C) up to a height of 12 cm and contained an invisible platform of 10 cm height and 8 × 8 cm surface area at the end of either the E or W arm. Each mouse had to perform 6 starts per day, alternating between N and S as a starting position in a semi-random fashion. If mice were started from N, the S arm was closed off; if mice were started from S, the N arm was closed off, thus turning the set-up into a functioning T-maze. For initial spatial memory acquisition training (= week 1) the platform was always located at the end of the W arm. In case of reversal learning (= week 2, **Experiment (I)**) the platform was located at the end of the E arm. Latency to reach the platform, wrong arm entries and wrong platform visits (entering the outer third of the arm opposite the platform containing arm) were recorded during training and later translated to Accuracy scores. A trial was deemed “accurate” if the animals swam directly to the platform without entering the wrong arm or returning to the start arm. An animal was deemed “accurate” if it performed at least 5 (out of 6) accurate trials per day (≥83.3%). The number of learners was calculated as the percentage of accurate performers per experimental group per day.

### Histology

Histopathological evaluation was done 37 days pi for **Experiment (I)** and** (II)**, and 11 days pi for **Experiment (III)** and** (IV)**. For **Experiment (I)** all animals survived the treatment (10 PBS : 10 SAVA). During **Experiment (II)** one SAVA-treated animal did not survive until the end, leaving 8 PBS: 8 ucAB: 7 SAVA-treated mice. For **Experiment (III)** two SAVA-treated mice and one PBS-treated animal did not survive until the end, leaving 13 PBS: 14 SAVA treated mice. During **Experiment (IV)** two SAVA-treated animals did not survive until the end, leaving 10 PBS: 14 SAVA-treated mice.

Generally, mice were perfused with 4% paraformaldehyde (PFA) in PBS + 0.1% glutaraldehyde (GA). Afterwards brains were carefully dissected out, post-fixed overnight in 4% PFA-PBS at 4°C and subsequently stored in 30% sucrose in PBS + 0.2% sodium azide (NaN_3_) at room temperature (ca. 21°C).

Next, 30 µm-thick coronal forebrain sections were cut with a freezing microtome resulting in a series comprising each 10th section. The sections were collected in 0.1 M Tris-buffered saline, pH 7.4 (TBS) containing sodium azide and were then stored at 4°C.

A first series of free-floating sections from all animals was applied to the concomitant immunofluorescence labeling of immunoreactivities for parvalbumin (as marker for a large subset of GABAergic neurons) and the vesicular glutamate transporter (VGLUT) 1. After extensive rinsing of the tissues with TBS, non-specific binding sites for subsequently applied immunoreagents were blocked with 5% normal donkey serum in TBS containing 0.3% Triton X-100 (NDS-TBS-T) for 1 h. The sections were then incubated overnight with a mixture of guinea pig-anti-parvalbumin (Synaptic Systems; 1:300 in NDS-TBS-T) and rabbit-anti-VGLUT1 (Synaptic Systems; 1:500). Following 3 rinses with TBS, immunoreactivities were visualized with a mixture of carbocyanine (Cy)2-conjugated donkey-anti-guinea pig IgG and Cy2-tagged donkey-anti-rabbit IgG (both from Dianova, Hamburg, Germany; 20 µg/ml TBS containing 2% bovine serum albumin = TBS-BSA) for 1 h.

For the detection of immunolesion-induced gliosis, a further forebrain series from all mice was primarily blocked with NDS-TBS-T for 1 h followed by incubation with a cocktail of guinea pig-anti-glial fibrillary acidic protein (GFAP; Synaptic Systems; 1:200 in NDS-TBS-T), affinity-purified rabbit-anti-ionized calcium adapter molecule 1 (Iba: Synaptic Systems; 1:400) and biotinylated *Solanum tuberosum* lectin (STL; Vector, Burlingame, CA, USA; 20 µg/ml) overnight. Rinsed sections were then immunolabeled with Cy2- and Cy3-coupled secondary antibodies as above, and lectin-binding sites were concomitantly revealed with Cy5-conjugated streptavidin (Dianova; 20 µg TBS-BSA) for 1 h. Finally, all sections were extensively rinsed with TBS, briefly washed with distilled water, mounted onto glass slides, air-dried and coverslipped with Entellan in toluene (Merck, Darmstadt, Germany). The omission of primary antibodies and biotinylated STL resulted in the expected absence of immuno- and lectin-histochemical labeling.

Pictures of immunofluorescence labeling were obtained with a confocal laser-scanning microscope 510 Meta (Zeiss, Oberkochen, Germany), using an argon laser (488 nm) for the excitation of Cy2, and two helium-neon lasers exciting Cy3 (543 nm) as well as Cy5 (633 nm). These band-pass (BP) filters were applied: BP 500–530 nm (Cy2), BP 565–615 nm (Cy3) and BP 654–718 nm (Cy5, infrared).

#### Semi-quantification of parvalbumin immunolabeling

For **Experiment (I)** the immunolabeled sections of 5 mice per group were analyzed regarding the number of PV+ neurons in infralimbic, prelimbic and cingulate cortex areas. For **Experiment (III)** + **(IV)** the sections of 6 mice per group were analyzed regarding PV+ cells for all cornu ammonis (CA) regions throughout the dHPC. Experimental groups were compared by means of consecutive coronal sections and PV+ neurons were pooled bilaterally per animal.

Picture acquisition was performed using a Keyence microscope BZ-9000 (Düsseldorf, Germany) and associated software. Statistical analysis of the collected data was carried out as described below.

### Statistical analyses

Behavioral and histological datasets were obtained blind to the treatment of the animals. They were analyzed by means of parametric (*t*-test or ANOVA followed by Tukey HSD *post-hoc* test given significant factorial interactions) or distribution (chi^2^) statistics using SPSS (IBM SPSS Statistics; New York, USA), STATISTICA for Windows (V 5.0 StatSoft, Inc., Tulsa, OK, USA; 1995) and GraphPad Prism™ (version 5.0; GraphPad Software Inc.; San Diego, CA, USA). Results were considered significant if *p* < 0.05. All results were plotted with GraphPad Prism™ (version 5.0; GraphPad Software Inc.; San Diego, CA, USA) as mean ± standard error of mean (Mean ± SEM).

## Results

### Experiment (I)—behavioral and histopathological consequences of long-term GABAergic depletion in prelimbic cortex

**Figure 1:**
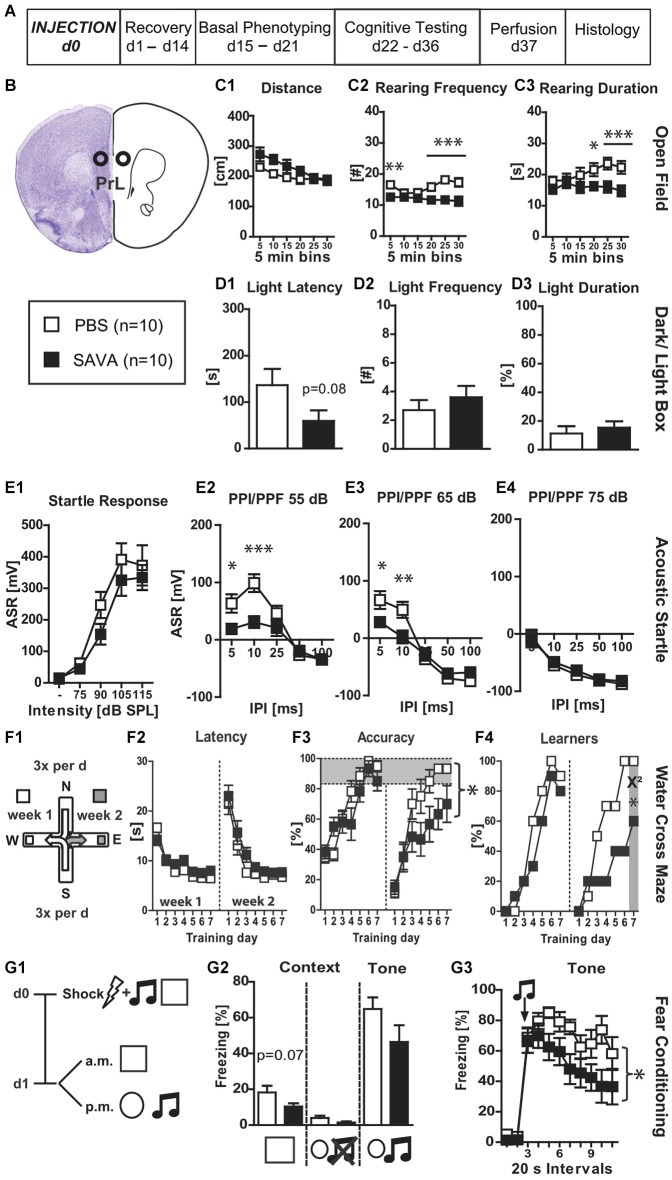
**Experiment I— behavioral consequences of long-term GABAergic depletion in prelimbic cortex (PrL)**: **(A)** timeline for Experiment **I**: stereotaxic PBS or SAVA injection at d0, recovery for 14 days, basal phenotyping (OF, D-L Box, ASR (I/O, PPI/PPF)) d15 to 21 post injection; cognitive testing (WCM, FC) d22 to 36 pi. Brains were collected after transcardial perfusion on d37. **(B)** Injection sites for Exp. **I**: PrL, bilateral, 0.5 µl each side (l 0.5; a-p +1.9; v 2.5 mm from Bregma); groups and sample size for Experiment **I**; **(C1–3)** OF behavior; **(D1–3)** D-L behavior ** (E1–4)** ASR as basic Input/Output curve and PPI/PPF curves after a 55, 65, 75 dB pre-pulse, respectively, for 5 different IPIs; **(F1)** basic schema of WCM; **(F2–4)** learning parameters for WCM; **(G1)** timeline for FC: tone-shock pairing on d0; testing for contextual and tone memory in conditioning and novel context on d1; **(G2)** comparison of contextual memory in the different contexts with and without tone presentation presented as freezing behavior over the course of 3 min observation periods (%); **(G3)** tone memory presented as freezing behavior (%) in novel context before and after presentation of tone. All data presented as mean ± SEM; ^*^p < 0.05, ^**^p < 0.01, ^***^p < 0.001 (Student’s *t*-test, chi^2^ test or ANOVA followed by Tukey HSD *post hoc* test). ASR = acoustic startle response; dB SPL = decibel sound pressure level; D-L = dark-light box; FC = fear conditioning; I/O = input/output; IPI = inter pulse interval; OF = open field; pi = post injection; PBS = phosphate-buffered saline; PPI/PPF = pre-pulse inhibition/facilitation; WCM = water cross maze.

**Experiment (I)** analyzed the effects of GABAergic depletion in the PrL (Figures 1A,B). SAVA administration did not affect the Distance traveled in the OF (Figure 1C1) but decreased both rearing frequency and rearing duration (Figures 1C2,3; RF: treatment × time: *F*_(5,90)_ = 5.67, *p* = 0.00014 and RD: *F*_(5,90)_ = 3.24, *p* = 0.0098). Furthermore, SAVA treatment caused a trend towards a decreased latency for entering the light compartment of the D-L box (i.e., slight decrease in anxiety related behavior; Figure 1D1; *p* = 0.08), without altering the frequency to enter the light compartment or the time spent in it (Figures 1D2,3). SAVA-treated mice showed unaffected acoustic startle-input/output responses (ASR-I/O; Figure 1E1), but displayed decreased pre-pulse facilitation (PPF) for pre-pulse intensities of 55 dB and 65 dB, but not 75 dB (Figures 1E2–4; 55 dB: treatment × inter-pulse interval: *F*_(4,72)_ = 5.36, *p* = 0.00078; 65 dB: treatment × inter-pulse interval: *F*_(4,72)_ = 8.02, *p* < 0.0001).

Regarding WCM performance, SAVA-treated mice did not show any differences during week 1 of training (i.e., ***acquisition***; Figures 1F1–4), but revealed a decreased accuracy during week 2 (i.e., ***reversal learning***; Figure 1F3; treatment × day: *F*_(6,108)_ = 2.73, *p* = 0.017). These treatment effects were also mirrored at the population level when comparing the number of accurate learners at the last day of each week (Figure 1F4; chi^2^ week 1 day 7: *p* = 0.5312; chi^2^ week 2 day 7: *p* = 0.025).

SAVA treatment resulted in a trend towards reduced contextual fear memory 24 h after shock application (Figures 1G1,2; *p* = 0.07) and a more rapid decrease in their freezing response over the course of the 3-min re-exposure to the conditioned tone (Figure 1G3; treatment × time: *F*_(10,180)_ = 2.09, *p* = 0.027).

Histopathological analyses (Figures 6A1–6) revealed a significantly decreased number of PV+ cells in the PrL (*p* = 0.0294; Figure 6A6) and anterior cingulate cortex (*p* = 0.0408; Figure 6A5) of SAVA treated animals. While we observed some loss of PV+ cells in the infralimbic cortex of SAVA-treated mice, the difference failed to reach statistical significance (data not shown; *p* = 0.21) and thus points to a limited dorso-ventral spread of GABAergic depletion.

**Figure 2:**
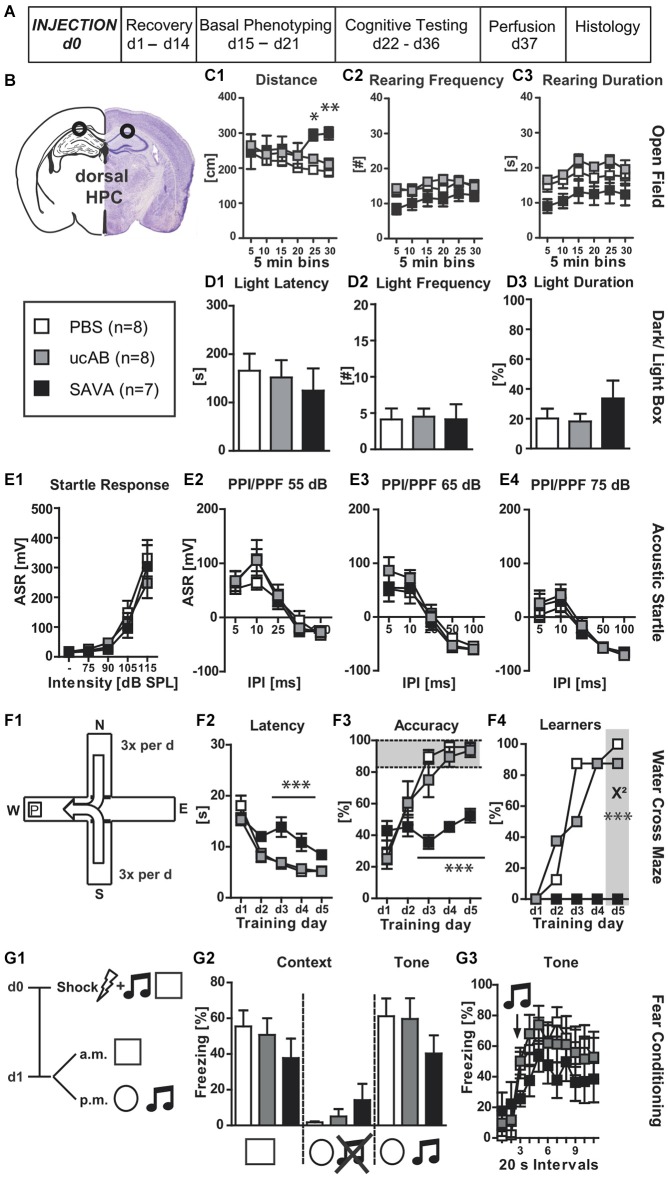
**Experiment II— behavioral consequences of long-term GABAergic depletion in dorsal hippocampus (dHPC)**: **(A)** timeline for Experiment **II**: stereotaxic PBS, ucAB or SAVA injection at d0, recovery for 14 days, basal phenotyping (OF, D-L Box, ASR (I/O, PPI/PPF)) d15 to 21 post injection; cognitive testing (WCM, FC) d22 to 36 post injection. Brains were collected after transcardial perfusion on d37. **(B)** Injection sites for Experiment **II**: dHPC, bilateral, 2µl each side (l 1.3; a-p −1.8; v 2.0 mm from Bregma); groups and sample size for Experiment **II**; **(C1–3)** OF behavior; **(D1–3)** D-L Box behavior;** (E1–4)** ASR as basic Input/Output curve and PPI/PPF after a 55, 65, 75 dB pre-pulse, respectively, for 5 different IPIs; **(F1)** basic schema of WCM; **(F2–4)** learning parameters for WCM; **(G1)** timeline for FC: tone-shock pairing on d0; testing for contextual and tone memory in conditioning and novel context on d1; comparison of contextual memory in the different contexts with and without tone presentation presented as freezing behavior over the course of 3 min observation periods (%); **(G3)** tone memory presented as freezing behavior (%) in novel context before and after presentation of tone. All data presented as mean ± SEM; ^*^p < 0.05, ^**^p < 0.01, ^***^p < 0.001 (chi^2^ test or ANOVA followed by Tukey HSD *post hoc* test; ucAB = unconjugated antibody; other abbreviations as in Figure 1).

**Figure 3:**
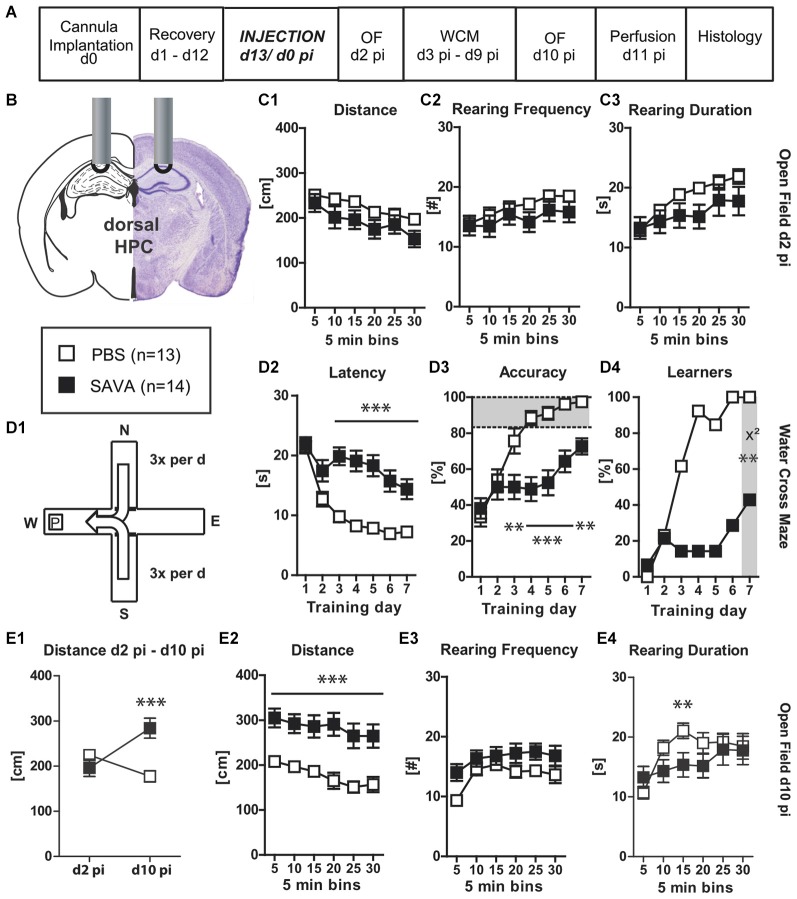
**Experiment III— behavioral consequences of short-term GABAergic depletion in dorsal hippocampus (dHPC)—*acquisition***: **(A)** timeline for Experiment **III**: stereotaxic cannula implantation at d0, recovery for 12 days, SAVA application = d0 pi; OF testing d2 pi + d10 pi, WCM training d3 pi until d9 pi, harvesting of brains after transcardial perfusion on d11 pi; **(B)** cannula position for SAVA **III**: dHPC, bilateral, l 1.3; a-p −1.8; v 1.0 mm from Bregma; injection of 0.5 µl PBS or SAVA per cannula; groups and sample size; **(C1–3)** OF behavior d2 pi;** (D1)** basic schema of WCM; **(D2–4)** learning parameters in the WCM; **(E1)** OF Distance d2 pi vs. d10 pi; **(E2–4)** OF behavior d10 pi All data presented as mean ± SEM; ^*^p < 0.05, ^**^p < 0.01, ^***^p < 0.001 (Student’s *t*-test, chi^2^ test or ANOVA followed by Tukey HSD *post hoc* test; abbreviations as in Figure 1).

**Figure 4:**
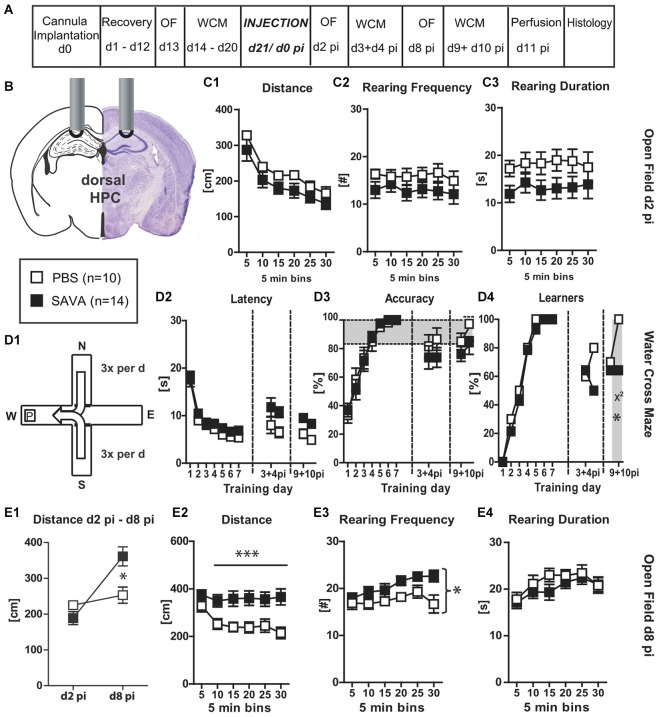
**Experiment IV— behavioral consequences of short-term GABAergic depletion in dorsal hippocampus (dHPC)—*recall***: **(A)** timeline for Experiment **IV**: stereotaxic cannula implantation at d0, recovery for 12 days, OF testing on d13, WCM training d14–20, SAVA application = d0 pi, OF testing d2 pi + d8 pi, WCM recall-training d3 + 4 pi and d9 + 10 pi, harvesting of brains after transcardial perfusion on d11 pi; **(B)** cannula position for SAVA **IV**: dHPC, bilateral, l 1.3; a-p −1.8; v 1.0 mm from Bregma; injection of 0.5 µl PBS or SAVA per cannula; groups and sample size; **(C1–3)** OF behavior d2 pi; **(D1)** basic schema of WCM; **(D2–4)** learning parameters in the WCM; **(E1)** OF Distance d2 pi vs. d10 pi; **(E2–4)** OF behavior d8 pi All data presented as mean ± SEM; ^*^p < 0.05, ^**^p < 0.01, ^***^p < 0.001 (Student’s *t*-test, chi^2^ test or ANOVA followed by Tukey *post hoc* test; abbreviations as in Figure 1).

**Figure 5:**
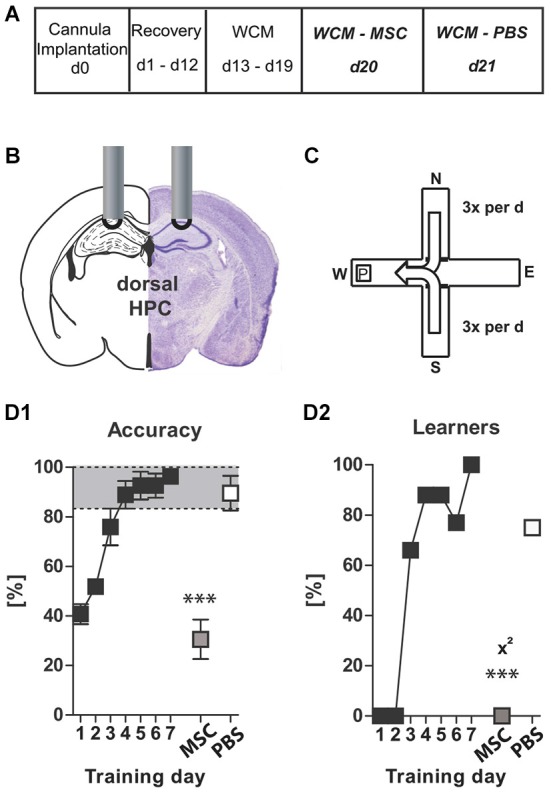
**Experiment V: Muscimol–Control**: **(A)** timeline for Experiment V: stereotaxic cannula implantation at d0, recovery for 12 days, WCM training d13–19, Muscimol treatment and WCM recall d20, PBS Treatment and WCM recall d21; **(B)** Cannula position for muscimol/PBS injections: dHPC, bilateral, l 1.3; a-p −1.8; v 1.0 mm from Bregma; injection of 0.5 µl MSC (125 µg/µl) or PBS per hemisphere; **(C)** basic schema of WCM; **(D1 + D2)** learning parameters for WCM. Data presented as mean ± SEM; ^***^p < 0.001 (chi^2^ test or paired Student’s *t*-test). dHPC = dorsal hippocampus; MSC = muscimol; PBS = phosphate-buffered saline.

**Figure 6:**
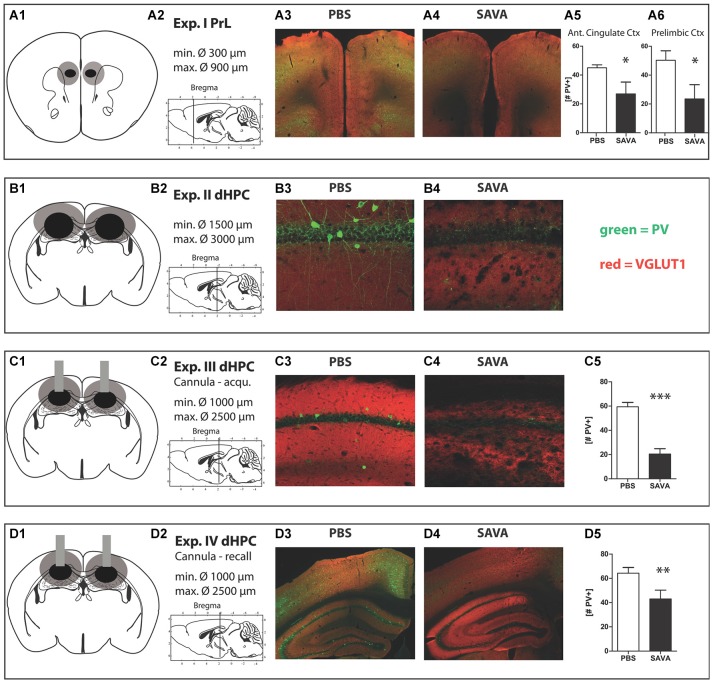
**Histology Experiment I through IV: (A1 + 2–D1 + 2)** respective location and extension of lesions for Experiment **I–IV**; **(A1–D1)** lesion extent: black = smallest occurring lesion, gray = biggest occurring lesion;** (A3)** representative image of prelimbic region of PBS-treated mouse;** (A4)** representative image of prelimbic region of SAVA-treated mouse;** (A5 + 6)** quantification of parvalbumin positive cells (PV+) in anterior cingulate cortex and prelimbic cortex, respectively;** (B3)** representative image of CA1 region of PBS-treated mouse;** (B4)** representative image of CA1 region of SAVA-treated mouse;** (C3)** representative image of CA1 region of PBS-treated mouse;** (C4)** representative image of CA1 region of SAVA-treated mouse;** (C5)** quantification of PV+ cells for Experiment **III** for all CA sub-regions;** (D3)** representative image of left dHPC of PBS-treated mouse;** (D4)** representative image of left dHPC of SAVA-treated mouse;** (D5)** quantification of PV+ cells for Experiment **IV** for all CA sub-regions; stainings: green = PV+; red = VGLUT1; Data presented as mean ± SEM; ^*^p < 0.05, ^***^p < 0.001 (Student’s *t*-test). CA = cornu ammonis; PBS = phosphate-buffered saline; PV = parvalbumin.

### Experiment (II)—behavioral and histopathological consequences of long-term GABAergic depletion in the dorsal hippocampus

**Experiment (II)** investigated the behavioral effects of GABAergic interneuron ablation via SAVA immunotoxin in the dHPC (Figures 2A,B). GABAergic neuronal loss in the dorsal HPC increased the distance traveled in the OF for the last 10 min of testing (Figure 2C1; treatment × time: *F*_(10,100)_ = 3.63, *p* = 0.0004). However, loss of GABAergic neurons in the dHPC had diverging effects on rearing behavior. Rearing frequency was not affected by immuno-lesioning (Figure 2C2), but the rearing duration was significantly decreased in SAVA-treated animals (Figure 2C3; treatment effect: *F*_(2,20)_ = 4.09, *p* = 0.0323). In contrast, anxiety-related behavior in the D-L (Figures 2D1–3), ASR-I/O (Figure 2E1) and PPI/PPF (Figures 2E2–4) were all unaffected by GABAergic neuronal loss in the dHPC.

When tested for spatial place learning abilities in the WCM (Figure 2F1), SAVA-treated mice showed marked impairments for all learning parameters (Figures 2F2–4): SAVA-treated animals displayed a higher latency to reach the platform (Figure 2F2; treatment × day: *F*_(8,80)_ = 5.12, *p* < 0.0001), and were less accurate in their performance (Figure 2F3; treatment × day: *F*_(8,80)_ = 7.46, *p* < 0.0001), which again also became evident at the population level when comparing the number of accurate learners between treatment groups at the end of training (Figure 2F4; chi^2^ day 5: *p* < 0.0001). Since none of the SAVA-treated animals had learned the location of the platform at the end of training, we refrained from assessing reversal-learning abilities.

Lastly, SAVA treatment of the dHPC failed to significantly affect contextual and auditory cued fear memory 24 h after shock application (Figures 2G1–3).

Histopathological analyses revealed a complete absence of PV+ from the dHPC (Figures 6B3,4; due to massive cellular loss statistics not applicable). However, despite precise injection locations SAVA-induced GABAergic lesions spread beyond the confines of the hippocampal formation, in particular affecting the posterior parietal cortex dorsal to the injection site.

### Experiment (III)—behavioral consequences of short-term GABAergic depletion in dorsal hippocampus; focus on spatial memory ACQUISITION

Given the massive cell-loss throughout the dHPC 37 days after SAVA application **Experiment (III)** investigated the short-term effects and thus the specificity of the effects following GABAergic depletion in the dHPC, with a particular focus on the acquisition of a spatial memory (Figures 3A,B). SAVA treatment did not affect the distance traveled nor the rearing behavior in the OF two days after its administration (Figures 3C1–3). However, ten days pi SAVA-treated animals displayed a drastically heightened distance traveled in the OF (Figures 3E1,2; Distance d2 pi *vs*. d10 pi treatment × day: *F*_(1,25)_ = 31.1914, *p* < 0.0001; Distance d10 pi treatment effect: *F*_(1,25)_ = 17.15, *p* = 0.0003) but still no changes in rearing behavior (Figures 3E3,4). Furthermore, WCM training beginning three days after SAVA administration (Figure 3D1) revealed a severe learning impairment for SAVA-treated animals across all learning parameters (Figures 3D2–4): SAVA-treated mice displayed a longer latency to reach the platform (Figure 3D2; treatment × day: *F*_(6,150)_ = 8.28, *p* < 0.0001), a decreased accuracy to swim directly to the platform (Figure 3D3; treatment × day: *F*_(6,150)_ = 7.63, *p* < 0.0001) and a reduced number of accurate performers at the end of training (i.e., Learners, Figure 3D4; chi^2^ day 7: *p* = 0.0012).

Histopathological analyses of Experiment **III** (Figures 6C1–5) revealed a vastly reduced number of PV+ cells in the CA regions of the dHPC in SAVA-treated animals (*p* < 0.0001; Figure 6C5), which once again additionally affected cortical regions.

### Experiment (IV)—behavioral consequences of short-term GABAergic depletion in dorsal hippocampus; focus on spatial memory RECALL

In order to determine whether GABAergic neuronal loss in the dHPC causes a general cognitive impairment or whether the observed effects were specific to the acquisition of a spatial memory, **Experiment (IV)** investigated the short-term consequences of GABAergic neuronal loss in the dHPC with particular focus on the recall of a spatial memory. Therefore, animals were equipped with guide cannulas aimed at the dHPC (similar to **Experiment (III)**), but were first trained in the spatial learning paradigm of the WCM until all of them had reached the accuracy criterion (Figures 4D1–4; Training day 1–7). Subsequently, animals were injected with SAVA or PBS and then repeatedly tested in OF and WCM (Figures 4A,B). Similar to **Experiment (III)**, SAVA treatment did not affect OF behavior on day 2 pi (Figures 4C1–3), but resulted in a significantly increased distance traveled on day 8 pi (Figures 4E1,2; Distance d2 pi vs. d8 pi treatment × day: *F*_(1,22)_ = 10.1958, *p* = 0.0042; d8 Distance treatment × time: *F*_(5,110)_ = 4.53, *p* = 0.0009) as well as a significantly increased rearing frequency (Figure 4E3; treatment effect *F*_(1,22)_ = 5.62, *p* = 0.027), but no changes in rearing duration (Figure 4E4). Re-testing in the WCM on day 3 + 4 pi revealed a treatment-dependent trend towards an increased latency (*F*_(1,22)_ = 4.08, *p* = 0.056) but no accuracy differences between groups (Figures 4D2,3). However, although not statistically significant, the number of learners in the PBS-treated groups increased from d3 to d4 pi (from 60% to 80%), whereas it decreased for SAVA-treated animals (from 64% to 50%). Re-testing on day 9 + 10 pi in turn, revealed a significant treatment effect on latency (*F*_(1,22)_ = 15.21, *p* = 0.0008) but only a treatment-dependent trend for performance accuracy (*F*_(1,22)_ = 3.55, *p* = 0.073). The number of learners per treatment group was nearly identical day 9 pi (PBS: 70%; SAVA: 64%; chi^2^ day 9 pi: *p* = 0.77), but again differed significantly on day 10 pi (PBS: 100%; SAVA: 64%; chi^2^ day 10 pi: *p* = 0.034; Figure 4D4).

Thus, initial recall performance was nearly identical for SAVA- and PBS treated mice at multiple testing time points pi. However, if the first recall day was followed by another day of training, PBS-treated animals improved once again in accuracy levels and number of accurate learners, whereas SAVA-treated animals stagnated or even decreased in WCM performance during re-acquisition, thereby replicating our findings of **Experiment** (**III**) regarding the (re-) acquisition-abilities of a spatial memory after GABAergic depletion in the dHPC.

Histopathological analyses (Figures 6D1–5) revealed a significantly reduced number of PV+ cells in the CA regions of the dHPC for SAVA-treated animals (*p* = 0.019; Figure 6D5). Similar to Experiment III SAVA-induced GABAergic lesions again also pertained to cortical structures close to the injection site.

### Experiment (V)—Muscimol–control

In order to demonstrate that the dHPC itself is involved in the recall of a spatial memory, a separate cohort of mice was equipped with guide cannulas aimed at the dHPC and then trained in the WCM (Figures 5A–D) until all of them had reached the accuracy criterion (d7, Figures 5C,D). One day later later mice were injected with muscimol (MSC; GABA_A_ agonist) into the dHPC, and 10 min thereafter their performance in the WCM was assessed. MSC injection resulted in a severely impaired WCM performance (Accuracy d7 vs. MSC: *p* = 0.0005; Learners d7 vs. MSC: *p* = 0.0001, chi^2^ test). Another 24 h later, following treatment with PBS, WCM performance was recovered (Figures 5C,D; Accuracy d7 vs. PBS: *p* = 0.1970; Learners d7 vs. PBS: *p* = 0.2794, chi^2^ test; Learners MSC vs. PBS: *p* = 0.0195, chi^2^ test). Thus, the dHPC is involved in the process of recalling a previously acquired memory; however, based on the results of **Experiment (IV)**, GABAergic interneurons of the dHPC are not necessary for this process.

In summary, we were able to demonstrate that (1) depletion of GABAergic neurons in the PrL **(Experiment (I))** results in decreased PPF (i.e., altered sensorimotor gating) and impairments in reversal learning (i.e., cognitive dysfunction), both of which are symptomatic hallmarks of schizophrenia and are widely replicated in animal models of this disease. Furthermore, we could show that depletion of GABAergic neurons from the dHPC causes (2) alterations in OF behavior (i.e., hyperlocomotion), the specificity and extent of which are dependent on the time point of testing in relation to SAVA treatment (**Experiment (II), (III) + (IV)**). In addition, we found that depletion of GABAergic neurons in the dHPC (3) impairs spatial memory acquisition (**Experiment (II)**). Moreover, we also observed a marked impairment in spatial learning abilities shortly after (i.e., 3 days) SAVA treatment (**Experiment (III)**), although injection volume as well as incubation period were drastically reduced compared to **(III)**, thus underlining the essential function of GABAergic hippocampal neurons for the acquisition of a spatial memory. In contrast, we found that (4) hippocampal GABAergic depletion does not affect the re-call performance of a previously acquired spatial memory (**Experiment (IV)**), implicating distinct GABAergic functions regarding the acquisition and the recall of a spatial memory.

## Discussion

Given the importance of GABAergic interneurons throughout the CNS and their often proposed major involvement in the disease etiology and progression of schizophrenia, this study aimed to further elucidate the behavioral consequences of GABAergic depletion in the PrL and the dHPC with regard to schizophrenia-related symptoms such as hyperlocomotion, altered sensorimotor gating and cognitive dysfunction.

We employed SAVA in order to deplete either the PrL or the dHPC from GABAergic interneurons (Antonucci et al., 2012). We confirmed the efficacy of the intervention by immunolabeling of PV+ neurons, which represent a large and important class of GABAergic interneurons (Celio, 1986; Caillard et al., 2000; Markram et al., 2004; Kubota, 2014) and have been previously implicated in schizophrenia-related disease etiology and symptom development (Gill and Grace, 2014).

Our data show that injection of the immunotoxin SAVA into the PFC did not affect the acquisition of a spatial memory, but selectively interfered with reversal learning (i.e., cognitive flexibility) and PPF of the ASR (i.e., sensorimotor gating). In contrast, SAVA injection into the dHPC severely impaired the acquisition of a spatial memory, independent of injection volume or incubation period. Strikingly, hippocampal SAVA treatment after memory acquisition failed to significantly affect the recall of a spatial memory. Furthermore, an incubation period of 10 days resulted in a markedly hyperactive phenotype, which was attenuated at later time points. In summary, SAVA treatment generates schizophrenia related positive symptoms (i.e., hyperlocomotion; deficits in sensorimotor gating) as well as cognitive deficits. The specificity of these effects is dependent on the target site and the duration of GABAergic depletion.

Although we were able to induce distinct and striking behavioral alterations with this approach, GABAergic depletion via SAVA injection has methodological limitations. For instance the hypothesized specificity, in particular regarding the injections in the PrL, could not be achieved. Rather, SAVA injection in the PrL resulted in GABAergic depletion throughout the medial prefrontal cortex (mPFC; *cf* Figure 6A). Furthermore, we cannot exclude that the observed phenotype after SAVA administration in **Experiment (I)** and **(II)** result from a cascade of neurodegenerative processes initiated by the selective lesioning of GABAergic neurons (Antonucci et al., 2012). However, such a cascade of secondary mechanisms and thus a steady increase in pathological severity are in fact hallmarks of many neurological disorders and our data might therefore indeed represent consequences of a pathological disease state. In fact, several recent publications have pointed out the neurodegenerative-like mechanisms observed for schizophrenic patients, in particular regarding abnormal gliosis (Black et al., 2004; Dwork et al., 2007; Pino et al., 2014) and have even linked schizophrenia to accelerated aging (Tang et al., 2009; Anthes, 2014). Therefore, while our approach might not allow us to specifically analyze the consequences of a purely GABAergic depletion, it effectively mimics a pathological state such as e.g., present in schizophrenic patients. Consequently, our work support a differential involvement of both HPC and PFC malfunction in schizophrenia as well as the time dependent symptom development following GABAergic manipulations.

There are several subclasses of GABAergic interneurons, which have been proposed to be differentially involved in generating a behavioral phenotype (Lewis, 2000; Markram et al., 2004; Kubota, 2014), however, our approach of SAVA injections does not allow for a distinction regarding their specific involvement in the observed behavioral effects. In order to determine the overall extent of the relative GABAergic neuronal loss for each target area, we chose to visualize parvalbumin containing (PV+) neurons, as they represent one of the largest subclasses of GABAergic interneurons and have been previously implicated in learning and memory-related processes (Caillard et al., 2000). Indeed, we observed significant PV+ loss after every SAVA injection. Nonetheless, given the strong and opposing effects after short-term SAVA incubation in the dHPC (i.e., memory acquisition vs. memory recall); we can conclude that SAVA injections distinctly affect the functionality of a local neuronal network and thus result in discreet behavioral alterations due to GABAergic depletion.

Remarkably, SAVA administration into the PrL/mPFC specifically affected PPF, but not PPI of the startle response. Most studies in animal models of schizophrenia employ IPI of ≥25 ms and, thus miss potential changes in PPF (Swerdlow and Geyer, 1998; Geyer et al., 2002; Plappert et al., 2004). In addition, ASRs with or without a given pre-pulse are not merely dependent on a possible pathology, but also on the genetic background of the employed animals as well as on the set-up itself (Paylor and Crawley, 1997; Plappert et al., 2004). Thus, the observed alterations in PPF, but not PPI, might result from a literal left-shift of the IPI-response curve. Alternatively the drastic interference of extensive GABAergic depletion in the mPFC via SAVA injection could also have revealed a novel sensorimotor-gating effect and therefore a *bona fide* PPF effect.

Previous work has implicated the mPFC and in particular the PrL in the expression of a fear response and has shown that decreased PrL activity resulted in decreased fear responses (Corcoran and Quirk, 2007; Piantadosi and Floresco, 2014). However, it has also been recently proposed that prelimbic and infralimbic areas of the mPFC exert opposing functions, with increased IL activity decreasing fear responses (Vidal-Gonzalez et al., 2006; Van De Werd et al., 2010; Ashwell and Ito, 2014). As mentioned above, our SAVA injections were not as specific to the PrL as originally hypothesized. Thus, IL GABAergic depletion could have resulted in increased IL excitatory output which would decrease amygdala output and result in decreased fear responses, which in turn could account for the observed accelerated decline in fear response to the auditory conditioned stimulus after SAVA application to the mPFC. Alternatively, we cannot entirely rule out that the reduced freezing response results from secondary loss of glutamatergic neurons of the PrL in consequence of the primary GABAergic lesion (Antonucci et al., 2012).

Different types of GABAergic neurons are known for their involvement in spatial learning (McNamara and Skeleton, 1993; Miettinen et al., 1993; Brucato et al., 1996; Iijima et al., 1996; Moser and Moser, 1998; Paulsen and Moser, 1998; Zilles et al., 2000; Bannerman et al., 2004; Ruediger et al., 2011; Buetfering et al., 2014). Our findings of intact acquisition but impaired reversal learning abilities following SAVA injection into the mPFC are concordant with the prominent role regarding cognitive flexibility ascribed to this brain structure (Euston et al., 2012). Correspondingly, the impairment of spatial learning abilities following SAVA injection into the dHPC is in line with our previous findings obtained by excitotoxic lesions of the hippocampus (Kleinknecht et al., 2012) as well as other works (Ruediger et al., 2011; Gilani et al., 2014). Nonetheless, we have to concede that the SAVA-induced GABAergic lesions were not entirely exclusive to the hippocampal formation and additionally pertained to cortical areas in close proximity to the injection site. In particular GABAergic loss and thus a malfunction of the posterior parietal cortex may have contributed to the deficits in spatial learning as observed in the present study (Harvey et al., 2012; Katsuki and Constantinidis, 2012; Myskiw and Izquierdo, 2012; Whitlock et al., 2012).

The importance of hilar interneurons for both the acquisition and the recall of a spatial memory has been previously reported (Andrews-Zwilling et al., 2012). However, our data provide first evidence for a distinction in the impact of GABAergic neurons beyond the hilar region on memory acquisition vs. memory recall. While SAVA-treatment three days before training in the WCM severely impaired spatial learning abilities, SAVA application in well-trained animals three days before the recall of a spatial memory failed to significantly impair the recall abilities. We controlled for a possibly transient involvement of the dHPC in the recall of spatial memories by treating a different cohort of mice with the GABA_A_ receptor agonist muscimol immediately before recall of spatial memory. Thus, we can conclude that although the dHPC is necessary for the recall of a previously learned platform position in the WCM, GABAergic neurons appear to be non-essential in this context.

In addition to the cognitive effects after SAVA administration at the level of the dHPC, we also observed a duration-dependent hyperlocomotor phenotype in these mice. An increased locomotor activity after the reduction of PV+ neurons in the dHPC has previously been reported in the context of rodent models for schizophrenia (Penschuck et al., 2006).

Given the shortened time-span between SAVA administration and behavioral assessment for Experiment III and IV, we concluded that the observed behavioral effects are most likely largely mediated by the GABAergic depletion in the dHPC itself and are representative of the early stages of the previously mentioned neurodegeneration-related cascade. However, aside from the consequences of SAVA administration on GABAergic interneurons, we also had to consider consequences of inflammation due to the injection/cannula implantation itself, as well as long-term consequences of GABAergic neuronal loss. It has been shown before that disrupting the integrity of skull and brain as a whole (e.g., via surgery) induces an inflammatory response (reviewed in e.g., Wang and Shuaib, 2002), which in turn can cause astrocytosis (Eng et al., 1992). An increased inflammatory response could also lead to alterations in behavior (Dantzer et al., 2008), and we did observe an increase in microglia activity in SAVA-treated animals compared to PBS treated animals (data not shown but previously published in (Antonucci et al., 2012)). However, the observed behavioral effects, in particular for experiments affecting the hippocampus, are so severe (i.e., ablation of spatial learning abilities) and also very specific to the time point of injection (acquisition vs. recall), that they are most likely not primarily based on an increased inflammatory response. This holds true in particular for SAVA-treated animals tested in **Experiment (III + IV)**. The animals in** (III)** displayed severe place learning impairments already on day five after SAVA administration, whereas animals in **(IV)** showed no effect on recall memory but a strong effect on locomotor activity.

Regarding long-term consequences of immunotoxin-induced loss of GABAergic neurons, Antonucci et al. (2012) previously reported a loss of CA1 pyramidal neurons 12 days after SAVA treatment. Since basal phenotyping for **Experiment (I) + (II)** only started on day 15 after SAVA administration, we cannot exclude that non-GABAergic neuronal populations were also successively affected and contributed to the observed phenotypes. However, **Experiment (III) + (IV)** resulted in clear and distinctive behavioral effects within 10 days of SAVA administration, which indicate a predominantly GABAergic driven effect (Antonucci et al., 2012). Furthermore, in terms of compensatory mechanisms, a reduction, albeit delayed, of excitatory neurons might in fact be beneficial for a functioning local neuronal network, particularly in the hippocampus, since inhibitory interneuron loss can lead to epileptiform seizures in animal models as well as in humans (de Lanerolle et al., 1989).

In conclusion, we demonstrated that GABAergic lesions result in distinct behavioral phenotypes specific to the target site and modulated by incubation time. We underlined the involvement of the medial prefrontal area in schizophrenia-like symptom development (sensorimotor-gating deficits and cognitive flexibility) and highlight a functional distinction of GABAergic neurons in the dHPC regarding spatial memory acquisition and memory recall: GABAergic neurons are necessary for the acquisition of a new spatial memory, but not for the recall.

While our approach did not enable us to distinguish discrete GABAergic neuronal circuits, we were able to simulate a pathological disease state and the consequences of prolonged GABAergic depletion. Therefore, our data provide new insights into behavioral alterations following GABAergic depletion, and in particular regarding the role of GABAergic neurons in the generation of positive schizophrenia-like symptoms such as hyperlocomotion and altered sensorimotor gating as well as the origin of cognitive deficits.

## Conflict of interest statement

The authors declare that the research was conducted in the absence of any commercial or financial relationships that could be construed as a potential conflict of interest.
